# New Senior Lecturer in Transplantation at Southmead

**Published:** 1991-12

**Authors:** Lesley Thomas


**NEW SENIOR LECTURER IN TRANSPLANTATION AT SOUTHMEAD**

Mr. Paul Lear was recently appointed as Senior Lecturer and Consultant in Transplantation Surgery at Southmead Hospital. He graduated from the Royal London Hospital in 1975 and most of his early training was in Nottingham. He spent two years in research work at the Harvard Medical School in Boston. He has a major research interest in intestinal transplantation.

Southmead currently carries out the majority of renal transplants in the South West Region in a programme which was initiated by the current Senior Surgeon 23 years ago. Since that time over 630 renal transplants have been carried out. Mr. Lear is keen to increase the number of renal transplants performed annually at Southmead and possibly to introduce combined pancreatic and renal transplantation as a treatment for diabetes in renal failure. He hopes that the smaller requirement for intestinal transplantation may be developed in future years.

His position is funded by generous support from the Southmead Health Authority, a small contribution from the University of Bristol and a major contribution for salary and research from the Bristol Kidney Research Fund.

Bristol Kidney Research Fund supports and sponsors locally based research into all aspects of renal disease including transplantation. Donations to support this cause could be sent to the Secretary,

Mrs. Lesley Thomas
Bristol Kidney Fund
Department of Renal Medicine
Southmead Hospital
Westbury-on-Trym
Bristol BS10 5NB.

